# Effects and neural mechanisms of different physical activity on major depressive disorder based on cerebral multimodality monitoring: a narrative review

**DOI:** 10.3389/fnhum.2024.1406670

**Published:** 2024-08-12

**Authors:** Jian Guan, Yan Sun, Yiming Fan, Jiaxin Liang, Chuang Liu, Haohan Yu, Jingmin Liu

**Affiliations:** ^1^Division of Sports Science and Physical Education, Tsinghua University, Beijing, China; ^2^Department of Sports, Beijing University of Posts and Telecommunications, Beijing, China; ^3^College of P.E and Sports, Beijing Normal University, Beijing, China; ^4^Department of Physical Education, Kunming University of Science and Technology Oxbridge College, Kunming, China; ^5^Department of Physical Education, China University of Geosciences, Beijing, China

**Keywords:** depressive disorder, exercise, brain plasticity, cognitive function, neural mechanis

## Abstract

Major depressive disorder (MDD) is currently the most common psychiatric disorder in the world. It characterized by a high incidence of disease with the symptoms like depressed mood, slowed thinking, and reduced cognitive function. Without timely intervention, there is a 20–30% risk of conversion to treatment-resistant depression (TRD) and a high burden for the patient, family and society. Numerous studies have shown that physical activity (PA) is a non-pharmacological treatment that can significantly improve the mental status of patients with MDD and has positive effects on cognitive function, sleep status, and brain plasticity. However, the physiological and psychological effects of different types of PA on individuals vary, and the dosage profile of PA in improving symptoms in patients with MDD has not been elucidated. In most current studies of MDD, PA can be categorized as continuous endurance training (ECT), explosive interval training (EIT), resistance strength training (RST), and mind–body training (MBT), and the effects on patients’ depressive symptoms, cognitive function, and sleep varied. Therefore, the present study was based on a narrative review and included a large number of existing studies to investigate the characteristics and differences in the effects of different PA interventions on MDD. The study also investigated the characteristics and differences of different PA interventions in MDD, and explained the neural mechanisms through the results of multimodal brain function monitoring, including the intracranial environment and brain structure. It aims to provide exercise prescription and theoretical reference for future research in neuroscience and clinical intervention in MDD.

## Introduction

Major depressive disorder (MDD) is a prevalent mental health disorder affecting approximately 280 million people worldwide, making it the third leading cause of disease burden globally. The World Health Organization (WHO) predicts that it will become the leading cause of disease burden by 2030(G.D.a.I. Collaborators, 2020). The global pandemic of COVID-19 has worsened many factors that contribute to mental health deterioration. This has led to a sustained increase in the prevalence of MDD, reaching 5% globally. The prevalence of MDD is high, affecting nearly one in 10 adults and up to 17% of adolescents in North America (Goodwin et al., 2022), about 7% in Europe (Arias-de la Torre et al., 2021), and as high as 34% in Asia-Pacific (Balakrishnan et al., 2022). The main symptoms of depression include a depressed mood, loss of energy, irritability, decreased appetite, and sleep disorders. In severe cases, MDD can damage both the central and peripheral nervous systems, affecting normal cognitive function and potentially leading to self-harm and suicidal ideation (Malhi and Mann, 2018). Approximately 71% of patients experience treatment-resistant depression (TRD), which can lead to irreversible psychological trauma if left untreated. This can cause significant burdens for individuals, families, and society. Timely intervention is crucial to prevent symptom exacerbation (Kulkarni et al., 2020).

Currently, drug therapy is the primary intervention for MDD. However, it is affected by side effects, addiction, and drug resistance, resulting in unsatisfactory overall efficacy. Additionally, it can cause a substantial increase in sedentary behavior, which can seriously affect the mental health and quality of life of patients (Almohammed et al., 2022). Cure rates for MDD remain low, with approximately 60% of patients worldwide not receiving adequate treatment (Mekonen et al., 2021). Therefore, some researchers have investigated alternative treatments for MDD. One such treatment is physical activity (PA), which has been shown to be an effective non-pharmacological intervention. PA not only reduces negative emotions and related symptoms, but also improves cognitive functioning and sleep quality. Its efficacy is comparable to that of drugs (Netz, 2017). However, research suggests that there may be variations in the physiological and psychological responses triggered by different types, durations, and intensities of PA (van Baak et al., 2021). For example, the American College of Sports Medicine (ACSM) categorizes PA into continuous endurance training (ECT), explosive interval training (EIT), resistance strength training (RST), and mind–body training (MBT) in the ACSM Guidelines for Exercise Testing and Prescription. training (ECT), explosive interval training (EIT), resistance strength training (RST), and mind–body training (MBT) (Thompson et al., 2022). Research suggests that EIT and RST have the most significant moderating effects on acute mood and sleep quality in patients with MDD (Singh et al., 2023). In contrast, ECT and MBT were more beneficial for maintaining exercise adherence and cognitive function (Schuch et al., 2016). To date, the number of studies investigating the dosage profile of different PAs in MDD interventions is still limited, and the associated neural and physiological underpinnings have not been elucidated.

This paper explores the quantitative and temporal effects of different PA on the antidepressant process. It compares the effects of various forms, durations, and intensities of PA in improving the related symptoms, cognitive functions, and sleep status of patients with MDD. Additionally, it analyzes the possible mechanisms and physiological bases of these effects. The aim of this paper is to provide effective recommendations and theoretical references for exercise prescription in future MDD treatment and intervention.

## Clinical symptoms and pathology of MDD

The primary symptoms of MDD are one of the leading causes of disease burden and disability worldwide and top the list of mental disorders (Dubovsky et al., 2021). The primary symptoms of MDD include persistent depressed mood, cognitive decline, autonomic dysfunction, and reduced quality of life. MDD includes bipolar II depression, mixed depression, agitated depression, atypical depression, melancholic depression, recurrent brief depression, minor depressive disorder, seasonal affective disorder, and other disorders (Benazzi, 2006). Research has shown that MDD is detrimental to both mental health and physical functioning. In terms of brain health, long-term MDD can lead to cognitive decline in executive function, memory, and inhibition, as well as a variety of neurodegenerative diseases, including Alzheimer’s disease, dementia with Lewy bodies, frontotemporal lobe dementia, and Parkinson’s disease (PE, 2015). Physically, MDD can lead to abnormalities in BMI, sinus rhythm, and immune function, as well as an increased risk of metabolic syndrome and cardiovascular disease (Vancampfort et al., 2015). In terms of mental health, MDD can be characterized by chronic depression, anxiety, or stress, as well as decreased self-esteem, self-efficacy, and self-control (LeMoult and Gotlib, 2019).

Regarding brain structure, MDD episodes are closely associated with a reduction in brain volume and changes in brain structure. Patients with MDD have been found to have abnormalities in the volume of frontal, temporal, thalamus, hippocampus, striatum, and amygdala structures (Koolschijn et al., 2009; Yaghmaei et al., 2009). Additionally, increased thickness of the temporal pole, right/left frontal gyrus, and paracentral region have been observed (Peng et al., 2015). There was a significant decrease in gray matter density in the hippocampus, amygdala (Espinoza Oyarce et al., 2020; Binnewies et al., 2021), and prefrontal cortex (PFC), as well as damage to white matter fiber tracts in frontal, parietal, and temporal regions. These changes were specifically reflected in the decline of cognitive function, memory, and executive function (Bora et al., 2012). For instance, a study discovered that while the whole brain volume of patients with MDD was not significantly different from that of healthy individuals, the hippocampal volume was significantly lower and the caudate nucleus was larger (Schmaal et al., 2016). This was particularly prominent in cases of chronic or recurrent depression and showed abnormalities in emotion regulation (Schmaal et al., 2016). One of the important hallmarks of MDD is a decrease in the volume of the striatum and paleostriatum (Wade et al., 2016). The patient’s deficits in interest were also contributed to by abnormalities in connectivity between the ventral striatum and several brain regions involved in reward processing (Pan et al., 2022). And this phenomenon is more severe in later life (Bora et al., 2012). Furthermore, research has shown that the severity of MDD is linked to decreased activity in the amygdala (Ferri et al., 2017). A study found that training to improve the amygdala’s response to positive memories significantly reduced depressive symptoms (Young et al., 2017).

Regarding the intracranial environment and plasticity, changes in the brain environment, certain nerve cells, neurotransmitters, and small molecules are closely linked to MDD. Studies have shown that patients with MDD have reduced neuron volume and glial cell density in PFC (Rajkowska et al., 1999). This is mainly due to the fact that MDD induces higher cortisol levels in the hippocampus, which in turn impedes neuronal growth in the brain (Lebedeva et al., 2018). Multiple studies have found that MDD is associated with imbalanced and reduced levels of neurotransmitters such as 5-hydroxy tryptamine (5-HT), norepinephrine (NE), brain-derived neurotrophic factor (BDNF), and dopamine (DA) in the brain. These imbalances lead to reduced nerve fiber conductivity and negatively impact an individual’s cognitive abilities and thought processes (Lima Giacobbo et al., 2019). Furthermore, there is evidence suggesting that the pathophysiology of MDD may be related to decreased levels of glutamate (Glu) metabolites in the medial frontal cortex and increased levels of acetylcholine (Moriguchi et al., 2019). Also based on the gamma-aminobutyric acid (GABA) hypothesis of MDD (Möhler, 2012), there are studies that have found lower levels of GABA within the cerebrospinal fluid and plasma of human and animal models of MDD (Streeter et al., 2020). On the other hand, increased peripheral and central inflammatory cytokines, acute phase protein (APP), and oxidative stress (OS) are also major markers of an abnormal intracranial environment in individuals with MDD (Troubat et al., 2021). Research has demonstrated that MDD is linked to elevated levels of inflammatory cytokines, including Interleukin-1 (IL-1), Interleukin-6 (IL-6), and Tumor Necrosis Factor-alpha (TNF-alpha), in the brain. These cytokines are known to regulate mood, appetite, and sleep (Goshen et al., 2008; Haapakoski et al., 2015). In addition, the involvement of OS in the pathogenesis of MDD is well established. The brain is particularly susceptible to OS due to its high oxygen and lipid content, but weak antioxidant capacity (Bhatt et al., 2020). OS also plays an important role in the pathophysiology of MDD through free radicals/nonradical molecules and reactive oxygen/nitrogen species (Vaváková et al., 2015).

## Dose characterization of PA in MDD interventions

### Differences in exercise patterns in relieving depression levels

#### Endurance continuous training

ECT is also known as aerobics exercise (AE) or moderate-intensity continuous training (MICT) that utilizes oxygen as a metabolic pathway to meet the body’s energy needs through oxidative phosphorylation. This type of exercise also leads to lower blood lactate levels (Hargreaves and Spriet, 2020). ECT is typically characterized by repeated moderate-intensity physical activity over a prolonged period, usually greater than 30 min. The intensity of ECT is typically in the range of 35–60% VO2max or 50–70% HRmax, which corresponds to 3–6 METs [metabolic equivalent = (oxygen consumption/resting oxygen consumption) × 3.5] (Mann et al., 2013). Research indicates that aerobic exercise, such as running, swimming, or cycling, is highly effective in improving MDD (Guszkowska, 2004).

Several large studies have confirmed the antagonistic and placebo effects of aerobic exercise on depressive symptoms. To explore whether a single session of ECT immediately alleviates depression levels, Martin et al. assessed the effect of a single 30-min walk on acute affective responses in patients with MDD. The exercise group demonstrated a significant reduction in negative affective responses and a substantial increase in positive affect levels following exercise compared to the passive control group. Light to moderate intensity walking activated higher levels of positive affective responses (Niedermeier et al., 2021). Studies have shown that short interventions can enhance the effect of a single ECT. Knubben et al. conducted a 10-day walking intervention on patients hospitalized for high levels of MDD. The exercise group showed a more significant reduction in both the Bech-Rafaelsen Melancholy Scale (BRMS) and the Center for Epidemiologic Studies Depression scale (CES-D) scores compared to the control group. Additionally, a higher percentage of patients in the exercise group experienced a reduction of 65 or more points on the BRMS (Knubben et al., 2007). Furthermore, additional studies have assessed interventions over extended periods. For instance, Matthew et al. conducted a 20-week trial comparing the effectiveness of ECT and modified rest and relaxation therapies for MDD. The final results indicated that the exercise group had lower Hamilton Depression Scale (HAMD) scores than the control group, and the depression remission rate was significant (Martiny et al., 2015). Similarly, Josine et al. randomly assigned 141 patients with MDD to either a medication or running exercise group. After 16 weeks, both interventions produced significant reductions in depression symptoms, with no significant difference in efficacy between the two groups. The study found that the running group showed significant physiological improvement compared to the control group. Additionally, the running group experienced weight reduction, improved blood pressure, optimized heart rate, and improved heart rate variability (Verhoeven et al., 2023).

**Figure 1:**
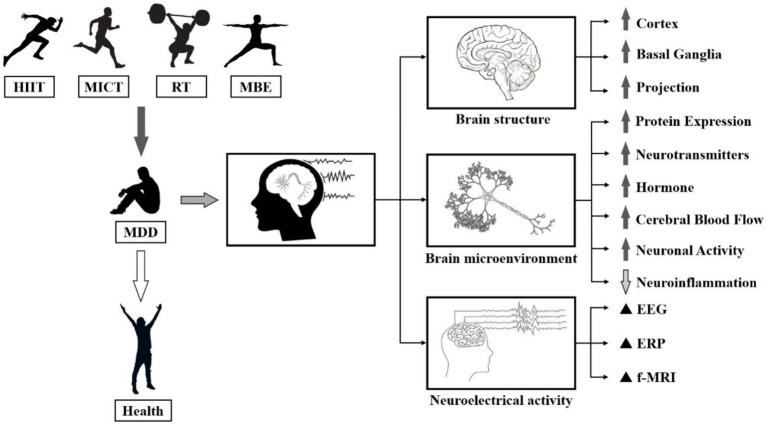
Biological manifestations of the effects of PA on MDD. Note: ⬆: Facilitation, ⬇: Repression, ▲: Regulation.

Although studies have tested the effectiveness of ECT, a large number of them have focused on comparing the duration and period of exercise as the main variables. In a 12-week intervention trial, Heinzel et al. compared the effects of high-intensity or low-intensity exercise on the alleviation of depressive symptoms in 120 patients with MDD. After the intervention, it was found that there was no significant difference between the groups in terms of Beck Depression Inventory (BDI) and HAMD, indicating that high-intensity exercise did not provide better relief from depression than low-intensity continuous exercise (Heinzel et al., 2022). In the 8-week trial conducted by Li et al., the MICT intervention was administered to 40 depressed patients at a frequency of 60 min, three times per week. This is a shorter-term example. The study found that patients’ HRSD-17 scores were reduced by 50% from baseline. Additionally, the Patient Health Questionnaire (PHQ-9) showed a significant decrease, while the Quality of Life Enjoyment and Satisfaction Questionnaire-Short Form (Q-LES-QSF) showed a positive improvement (Li et al., 2023). To better characterize the temporal effects of ECT on MDD, Bellón included 18 studies in a systematic review that assessed 1,737 subjects from 8 countries and found that at least 4 weeks of ECT significantly reduced depression scores in patients aged 18–64 years. Meanwhile, selective prevention (i.e., people exposed to risk factors for depression) was associated with lower efficacy of ECT in Asian (Bellón et al., 2021). In conclusion, numerous studies have confirmed the remarkable effectiveness of ECT, AE, and MICT. This type of exercise has positive effects on regulating negative emotions, increasing self-confidence, and improving mental health, which are particularly important for individuals with MDD who struggle with depression (Figure 1).

**Figure 2:**
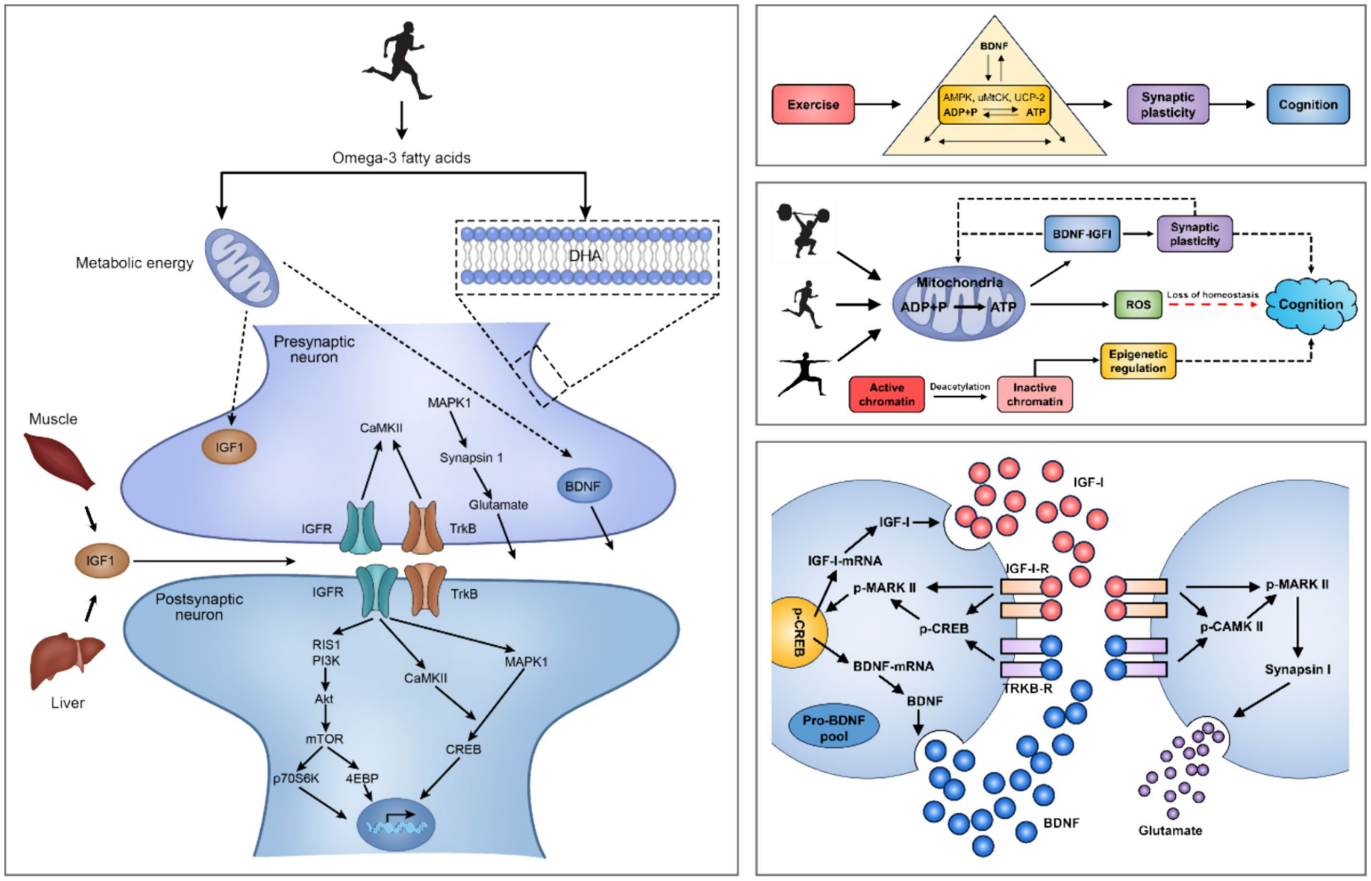
Mechanisms of exercise regulation of the intracerebral environment and small molecules in the brain. Note: Brain-Derived Neurotrophic Factor (BDNF), Insulin-Like Growth Factor 1 (IGF-1), IGF-1 Receptors (IGF-1-R), MAPK-Activated Protein Kinase 1 (MARK I), MAPK-Activated Protein Kinase 2 (MARK II), Phosphorylation of MARK II (p-MARK II), Calmodulin Dependent Protein Kinase II (CaMK-II), Insulin Like Growth Factor Receptors (IGFR), Tropomyosin-Related Kinase B (TrkB), TrkB Receptors (TrkB-R), Ras-Induced Senescence 1 (RIS1), Phosphoinositide 3-Kinases (PI3Ks), Mechanistic Target Of Rapamycin (mTOR), 70 kDa Ribosomal S6 Kinase (p70S6K), eIF4E-Binding Protein (4EBP), cAMP-Response Element Binding Protein (CREB), Phosphorylation of CREB (p-CREB). PA induces hippocampal production of IGF-1 and enhances hippocampal synaptic plasticity through BDNF downstream signaling mechanisms, including CAMK II, MARK I and p-MAPK II signal cascades. The interaction of IGF-1, BDNF mRNA and pro-BDNF protein occurs at the postsynaptic membrane.

Numerous studies have investigated the neural mechanisms of ECT and its effects on MDD. Evidence suggests that MDD is linked to elevated levels of inflammatory factors in the body and brain. However, ECT has been shown to decrease the secretion of pro-inflammatory cytokines, including IL-6 and tumour necrosis factor alpha (TNF-α) (Shih et al., 2013). Meanwhile, the decline of cognitive ability and neuroplasticity in patients with MDD poses a significant threat to their health. It has been widely demonstrated that ECT stimulates the secretion of benign small molecules in brain, including DA, NE, 5-HT, and endorphins, and significantly increases neuroplasticity in MDD patients (Ayari et al., 2023). Individuals’ intracranial release of BDNF after high-volume sustained exercise can repair the symptoms of hippocampal atrophy, which is believed to be the main reason why ECT improves cognitive function in patients with MDD (Hendrikse et al., 2022). In addition to affecting the nervous system, ECT can reduce the release of hormones, glucose, and oils that occur during a depressive state. It also improves the adrenal medulla’s ability to secrete catecholamines, which can help alleviate depressive symptoms (Duncan et al., 1985). Overall, ECT has a significant positive impact on improving physiological indicators and internal mechanisms in patients with MDD and is therefore often recommended as part of a comprehensive treatment program (Figure 2).

#### Explosive interval training

EIT is typically categorized as vigorous physical activity (VPA), with high intensity. >80% of maximum heart rate, 70% of maximum oxygen uptake, and more than 6 METs. During EIT, the body consumes energy substances such as creatine phosphate and glycogen through the anaerobic energy supply system, which includes the phosphagen and glycolytic systems, to supply sufficient ATP to meet the body’s needs. When engaging in EIT, the human body continuously consumes energy sources such as phosphocreatine and glycogen through the anaerobic energy supply system, including the Phosphagen System and Glycolytic System. This process supplies sufficient ATP to meet the body’s energy demand for a short period of time. However, it also results in a higher level of lactic acid. Numerous studies have investigated the efficacy differences between EIT and other forms of exercise in treating MDD. One of the most representative forms is High-Intensity Interval Training (HIIT) or Sprint Interval Training (SIT). The exercise format of HIIT, SIT usually refers to high intensity sprints of 80–95% HRmax/70–85% VO2max for 30s to 2 min, interspersed with low intensity intermittent recovery of 55–65% HRmax/40–55% VO2max for 1 min 30s to 4 min, during which MET levels can vary from 6 to 15 METs (Mann et al., 2013). Due to its time efficiency and greater impact on cardiorespiratory fitness, HIIT has been extensively researched in comparison to MICT regarding its effects on psychological well-being, physical fitness, and exercise enjoyment for both healthy individuals and those with MDD.

Several lines of evidence suggest that EIT has a positive moderating effect on the mental health and emotional state of people with MDD. Matthew et al. found through a systematic evaluation of multiple studies that the majority of patients produced favorable affective responses after a single session of EIT compared to a blank control group, and this effect was consistent across different types of affective states, exercise environments (Self-Selected/Imposed/Outdoor/Indoor), and participant characteristics (Bourke et al., 2022). Rebecca et al. conducted a comprehensive meta-analysis of 85 studies, which included a large number of people of all ages, as well as overweight and metabolically disordered patients. The results showed that HIIT intervention was effective compared to an inactive control group across all populations. HIIT at a frequency of at least 2 times/week for 2 weeks or more reduced the risk of depression, anxiety, other negative mood factors, and MDD. HIIT improved cardiorespiratory fitness, body composition, blood glucose and lipid concentrations, arterial compliance and vascular function, cardiac function and reserve, markers of inflammation in the brain and body, exercise capacity, and muscle mass significantly more than other treatments. No acute injuries were reported in any of the studies, and the average adherence rate was over 80% (Martland et al., 2020).

**Figure 3:**
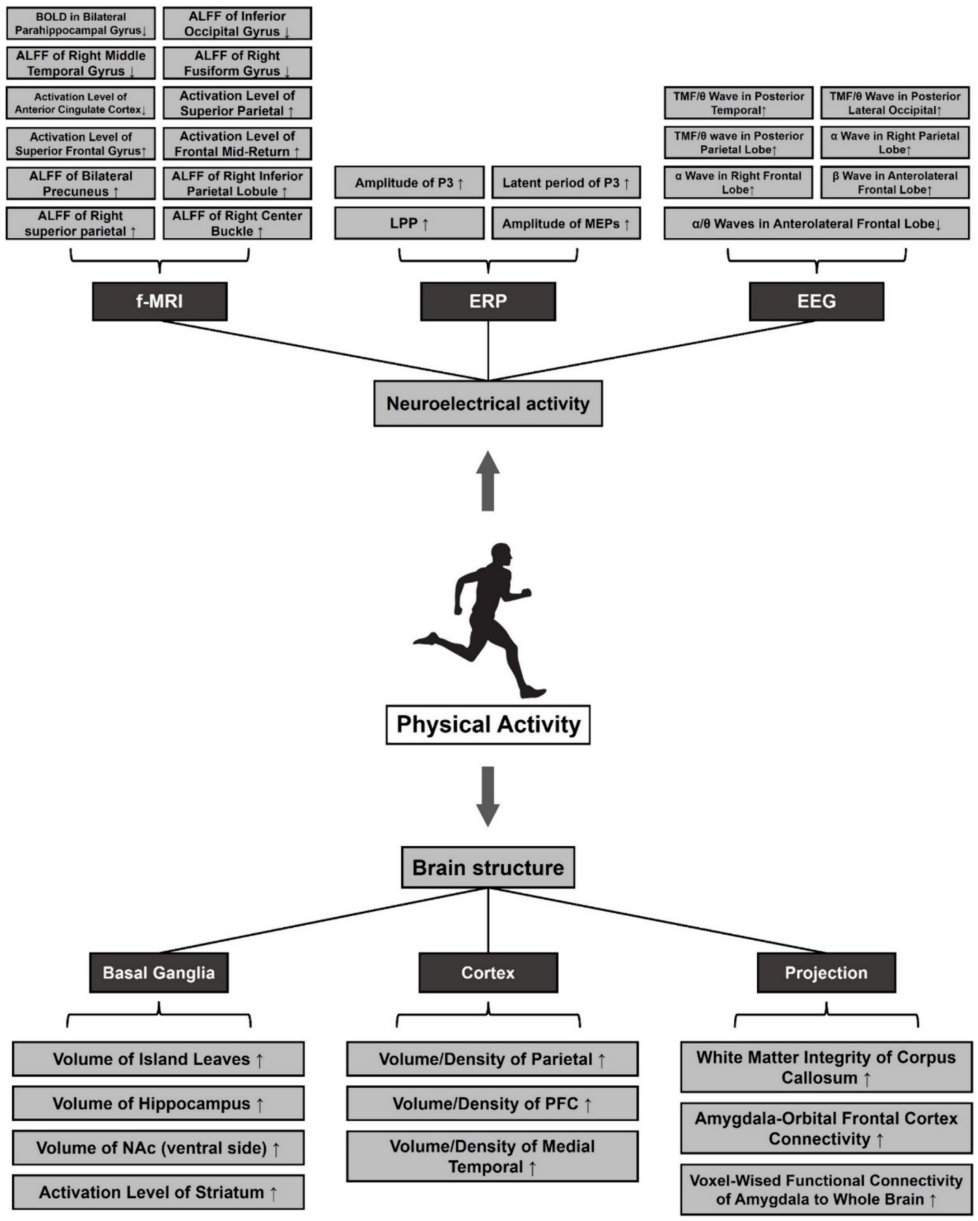
Effects of pa on structural plasticity and neuroelectrical activity of the brain in MDD. Note: ⬆: Facilitation, ⬇: Repression. Amplitude of Low-Frequency Fluctuations (ALFF), Late Positive Potential (LPP), Motor Evoked Potentials (MEPs), Total Mean Frequency (TMF), Nucleus Accumbens (NAc), Prefrontal Cortex (PFC).

Although EIT has been shown to promote mental health, it is still inconclusive whether its effects are superior to those of other PA. Alice et al. conducted 12-week AE and SIT interventions for patients with MDD and found that BDI scores of the subjects in both groups were significantly reduced. However, there was no significant difference between the groups, although SIT saved time and achieved the same effect as AE. There was no significant difference between the groups (Minghetti et al., 2018). The results may be attributed to the short intervention period. Generally, the duration of exercise can cause differences in individual physiological and psychological indicators. Markus et al. obtained similar results through a 35-week experiment. They divided 50 MDD patients into two groups including SIT and ECT, compared the two groups of patients in the after-training period for changes in indicators of positive affective response, self-perceived health, cardiorespiratory health, and level of depressive symptoms. Finally, both groups showed improvement in all metrics at the end of the intervention, except for autonomic exercise motivation, which did not significantly change from baseline. However, there were no significant differences between the groups, indicating a significant time effect. However, the ECT group was slightly more likely to reach the recommended level of physical activity after the intervention compared to the SIT group (Gerber et al., 2018). This may be due to the fact that ECT has a simpler training format and delivery conditions than SIT and a lower barrier, making it more suitable for first-time exercisers to develop exercise adherence (Figure 3).

**Figure 4:**
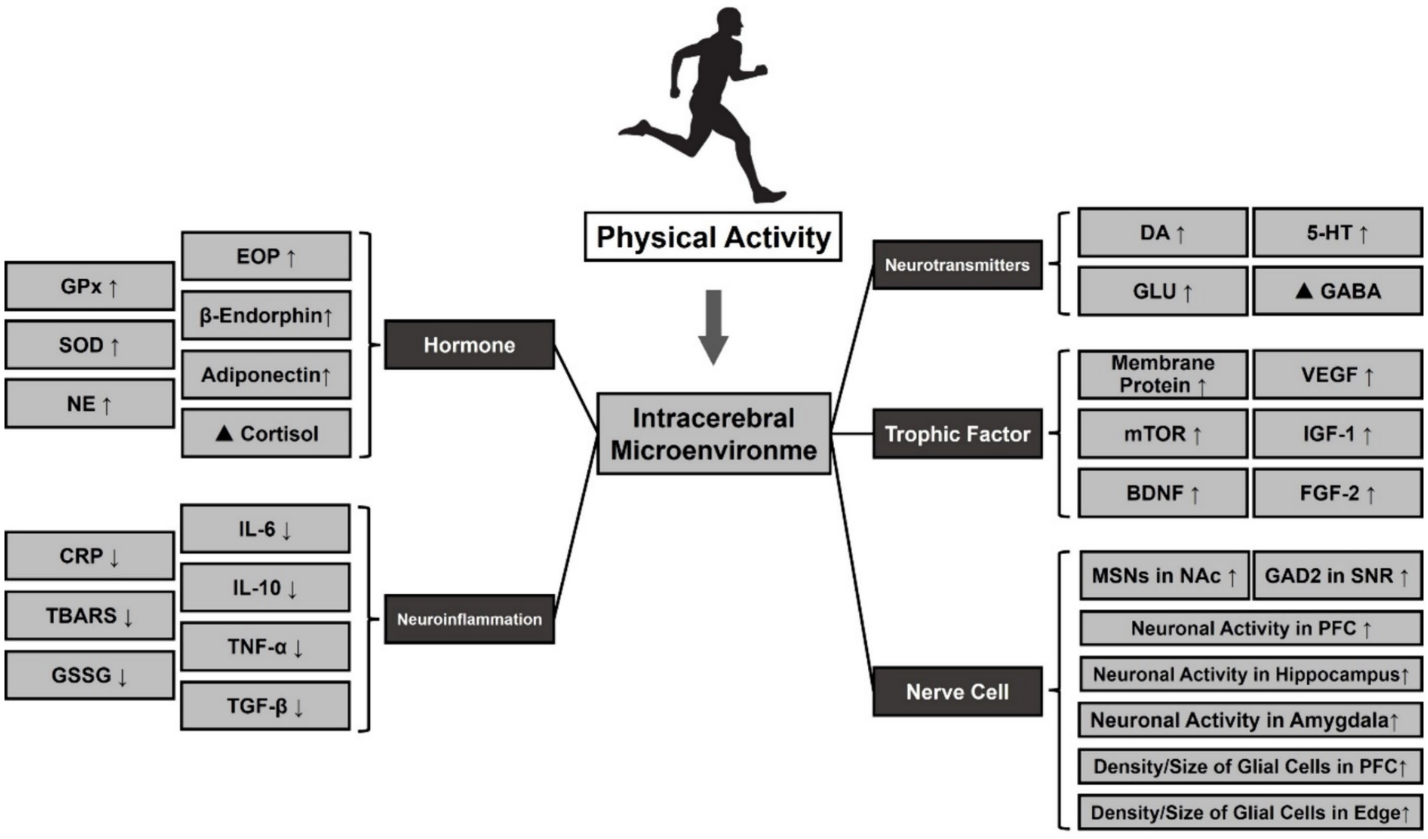
Effect of PA on the release of neurotransmitters and small molecules in MDD. Note: ⬆: Facilitation, ⬇: Repression, ▲: Regulation. Glutathione Peroxidase (GPx), Oxidative Stress (SOD), Norepinephrine (NE), Dopamine (DA), 5-Hydroxytryptamine (5-HT), Glutamic Acid (Glu), γ-Aminobutyric Acid (GABA), Vascular Endothelial Growth Factor (VEGF), Mammalian Target Of Rapamycin (mTOR), Insulin-Like Growth Factor 1 (IGF-1), Brain-Derived Neurotrophic Factor (BDNF), Fibroblast Growth Factor 2 (FGF-2), Medium Spiny Neurons (MSNs), Nucleus Accumbens (NAc), Glutamate Decarboxylase 2 (GAD2), Substantia Nigra Pars Reticulate (SNR), Prefrontal Cortex (PFC).

However, other studies have shown that EIT is more effective than other PAs for MDD. For example, Björg compared the efficacy of conventional medication (310), low-intensity ECT (106), moderate-intensity ECT (105), and EIT (99) in a one-year randomized controlled trial of 620 patients aged 18–67 years with MDD. The results showed that during the first 2 months, conventional medication significantly reduced patients’ scores on the Montgomery-Asberg Depression Rating Scale (MADRS). However, starting at 5 months, the three exercise groups outperformed the medication group in intervening on depressive symptoms. At the end of 1 year, the EIT group not only had the lowest MADRS score, but also the highest quality of life score based on the MOS item Short Form Health Survey (SF-36). At the same time, adherence to low-intensity ECT was the highest. This suggests that EIT improves the effectiveness of rehabilitation in MDD patients (Helgadóttir et al., 2017). To test this claim, Yolanda divided 67 COVID-19 matched adults into two groups, administered the HIIT and MICT, and assessed differences in anxiety, stress, and resilience using the BDI, State–Trait Anxiety Inventory (STAI), Perceived Stress Scale (PSS-10), Connor-Davidson Resilience Scale (CD-RISC) to assess differences between the two groups of subjects in anxiety, depression, stress, and mental toughness. The results showed that after 40 weeks of intervention, stress and anxiety symptoms were significantly reduced in both groups of subjects. However, the HIIT group had lower levels of depression and better mental toughness than the MICT group (Borrega-Mouquinho et al., 2021). To validate the temporal effect profile of HIIT, Nicole demonstrated in a systematic review of 366 patients with MDD that at least 4 weeks of HIIT significantly reduced depressive symptoms such as suicidal ideation and behavior, negative mood, and limitations in physiological functioning, and that 8 weeks of HIIT was superior to MICT in improving life satisfaction, social functioning, cognitive ability, and self-control, and there was no difference in adherence. MICT was superior to HIIT in improving life satisfaction, social functioning, cognitive ability, and self-control, and there was no significant difference in adherence (Korman et al., 2020). In response to the controversies in the aforementioned studies, Rebecca systematically summarized the mental health outcomes of HIIT based on her previous research. The results showed that both HIIT and MICT were effective in terms of acute mental health changes. However, there was no significant difference between the two groups, and only one study reported a more significant outcome for HIIT. Regarding long-term mental health changes, HIIT demonstrated greater reductions in anxiety levels between 3 months and 5 years. Additionally, HIIT showed moderate improvements in depression, anxiety, and psychological stress, but not superior to MICT (Martland et al., 2022). In conclusion, the available evidence suggests that there is no significant difference between HIIT and MICT in terms of improving mental health. However, it is important to consider the heterogeneous results of a small number of studies. Therefore, further research is needed to examine the temporal relationship between exercise intensity in MDD interventions (Figure 4).

So far, the superiority of EIT in MDD intervention is still inconclusive, and the heterogeneity of results from different studies is large. Therefore, this study explains the phenomenon from the perspective of neural mechanisms. EIT stimulate the release of neurotransmitters such as DA, NE, and BDNF, activate the growth of new neurons, and increase inter-synaptic communication, resulting in an increase in parasympathetic activity. This not only improves an individual’s mood and attention span but also decreases their resting heart rate, increasing their cardiac reserve (Zimmer et al., 2016; ROSS et al., 2019; Shirvani et al., 2019). ECT may also promote the secretion of such transmitters, but to a lesser extent. In the endocrine system, it has been found that acute EIT elevates cortisol and testosterone secretion levels to a greater extent than ECT (Dote-Montero et al., 2021). Under certain circumstances, moderate cortisol secretion can enhance the body’s ability to adapt. However, if cortisol remains elevated for an extended period, it can negatively impact the brain and nervous system. EIT helps the body regulate its levels and concentrations more effectively. From a circulatory perspective, the EIT process is typically accompanied by a rapid increase in blood flow and oxygen demand. If the body’s needs are not met within a short period of time, this can result in insufficient blood supply and oxygen deficit (Hill et al., 2008). ECT only accelerate blood flow and do not cause these conditions. Therefore, EIT results in a more significant increase in the amount of oxygen in the brain, especially after a sustained period. Overall, EIT has been shown to improve depressive symptoms, cognitive function, and brain plasticity in patients with MDD, but the specific dosage needs to be adjusted according to individual physiological factors and subjective readiness, such as HIIT or SIT.

#### Resistance strength training

Resistance strength training (RST) is a type of exercise that involves working against resistance to enhance muscle strength and volume. RST typically includes weights, resistance bands, or bodyweight exercises like push-ups or squats (Westcott, 2012). In general, the energy supply system of RST heavily relies on the body’s anaerobic energy system. This system uses stored energy in the form of ATP and phosphocreatine to provide energy for short bursts of intense activity. As the exercise continues, the body also relies on glycogen stored in the muscles to provide energy. Acute or prolonged Resistance strength training not only boosts metabolic levels and slows down the body’s aging process, but it can also effectively reduce negative emotions such as anxiety and depression (Hill et al., 2008; McLeod et al., 2019). Compared to MICT/HIIT, there are fewer MDD studies related to the design of RST. However, evidence suggests that RST can be an effective tool on its own or in combination with medication, and can have a positive effect on interventions for depression.

Currently, although there are few studies on the relationship between MDD and RST, evidence suggests that RST can be an effective tool, either alone or in combination with medication. For instance, Joseph et al. conducted a 12-month intervention in which 50 MDD male patients were randomly assigned to an RST group or an attention-training group. Results showed that at the end of the intervention, the RST group not only had significantly lower scores on the Quick Inventory of Depression Symptomology (QIDS) than the attention training group, but also had additional improvements in body composition, including muscle density, adiposity, and BMI (Ciccolo et al., 2022). To compare the effects of RST with other exercises on MDD, Helena extended the resistance strength training to older adults and compared the effects of 12 weeks of ECT and RST on a small sample of older adults with MDD (*n* = 27) using HAMD. The final results showed that both ECT and RST significantly reduced depression levels in older adults by approximately 50%. However, there was no significant difference between the two groups (Moraes et al., 2020). Krogh found similar results in a 12-month cohort study. After comparing the differences in the effects of MICT and RST on mental status and motor function in patients with MDD, he found that from month 4, both groups showed a significant decrease in HAMD and Hamilton Anxiety Scale (HAMA) scores and an increase in Mindful Attention Awareness Scale (MAAS)-based positive mindfulness. At the end of 12 months, the mental status of patients in both groups improved, and although there was no significant difference between the groups, the RST group had better strength qualities and BMI than the MICT group (Krogh et al., 2009). To characterize the timeliness of RST interventions for MDD, Barahona found through a systematic evaluation involving 768 patients with MDD that the effect of RST on improving depression levels was significant from at least 10 weeks and was accompanied by reductions in negative emotions such as anxiety, stress, sadness, and hostility, and increases in self-esteem, self-efficacy, and self-control (Barahona-Fuentes et al., 2021). The above results suggest that RST has similar effects on improving depressive symptoms and mental health in MDD patients as ECT and EIT, but the improvement effects on physiological functions, including body composition, strength quality, and exercise capacity, are more significant. Meanwhile, the effective period of RST is longer than other exercises, so it may not be conducive to the development of exercise adherence.

Although there is insufficient evidence to confirm that RST is superior to other exercises in improving MDD, RST is superior in improving patients’ motivation to exercise. For example, core symptoms of patients with MDD include a lack of interest and motivation, which in adults can lead to phenomena such as difficulty going to work on a daily basis or a low rate of return to work. For this reason, the mental health of workers in enterprises has long been a major concern of the WHO and international trade union organizations (Evans-Lacko and Knapp, 2016). Some studies have shown that RST is an effective modality that can significantly improve an individual’s level of depression and motivation to exercise. Karen et al. conducted a study on the return-to-work attendance of 5,996 patients with MDD after receiving usual care, cognitive therapy, medication, combined psychological and medication, and RST interventions. The study found that supervised RST significantly reduced sickness absence in patients with MDD and was superior to ECT and MBT (Nieuwenhuijsen et al., 2020). Meyer explained the superiority of RST in improving motivation to exercise in MDD patients from a psychological perspective in a 26-week cohort study. He found that at the end of the experiment, the RST group not only had lower levels of self-reported and clinically identified depression, but also had higher exercise adherence than the RCT control group. To explain this phenomenon, they reported exercise adherence at weeks 8, 16, and 24 and found that it increased from 90 to 93 and 98% in the RST group, respectively, and was accompanied by higher middle cerebral artery (MCA) blood flow velocity, electrical conductivity, and muscle circumference. No significant changes were observed in the RCT group. In contrast to the RST group, the RST group used a progressive training regimen, i.e., the number of sets and intensity of the training gradually increased as the individual’s physical fitness changed, thus triggering the Hawthorne effect in the patients, i.e., individuals tended to be willing to continue to persevere and increase the level of difficulty when they performed a task that was within their ability range and became motivated. In the RST group in this study, patients initially performed only a few sets of straight arm flexion and extension, and due to the specificity of RST, significant muscle hypertrophy was observed at the end of each training session. Later, when the training intensity was gradually increased, the “muscular hypertrophy” induced by the strength training became more significant, thus motivating the patients to complete the entire training program involving multiple movements at once, which is also considered to be the main reason why RST can more effectively stimulate the exercise motivation of patients with MDD (Meyer et al., 2024). Despite the significant benefits of RST in improving exercise adherence, the number of studies of RST in MDD is still very limited due to the large space, equipment, and technician requirements for implementation.

From a neural mechanism perspective, the antidepressant effects of RST may result from various effects on different aspects of the patient’s nervous system, some of which are similar to ECT and others that are different. Studies have shown that RST also increases the positive release of certain transmitters and small molecules in the brain, similar to other exercises, which in turn promotes neuronal growth. There is evidence that RST modulates plasticity changes in the Central Nervous System (CNS) and improves cognitive functioning (Chow et al., 2021). For instance, Majid discovered that administering RST to college students with MDD for 10 weeks resulted in a decrease in C-reactive protein, an increase in plasma levels of 5-HT and NE, and a moderate decrease in cortisol levels. These changes in monoamine transmitters were strongly associated with exercise-induced improvements in MDD (Khorvash et al., 2012). Not only that, but it has also been shown that prolonged RST is associated with a decrease in inflammatory factors in the brain, such as IL-6, TNF-alpha, and C-reactive protein (CRP), which are involved in the immune system’s or the liver’s response to inflammation, respectively. Elevated levels of these factors have a direct impact on the risk of depression, and regular RST reduces the secretion of these substances.

#### Mind–body training

Mind–body trainings (MBT) are physical activities designed to improve the connection between the mind and body, promoting relaxation, stress reduction, and positive thinking. MBT combine physical movement, breathing techniques, and mental focus to achieve these results. MBE, such as Tai Chi, Yoga, Qigong, or Mindfulness meditation programs, are characterized by a focus on breathing and positive thinking. These exercises often emphasize breath control and mental focus (Stringer, 2021). This exercise type is typically low to moderate intensity, with an intensity range of 50–70% HRmax/30–50% VO2max or 2–3 METs. It does not require a high level of physical exertion and is therefore suitable for individuals of all fitness levels. This type of exercise is widely popular and non-competitive (Saeed et al., 2019). Numerous physical and mental exercises involve whole-body movement and promote flexibility, balance, and coordination. To date, an increasing number of studies have shown that physical and mental exercise can effectively alleviate symptoms of depression.

A significant body of evidence suggests that MBT can be used as an adjunctive therapy to improve depressive symptoms in people with MDD and in various populations. In a meta-analysis, Ashok et al. evaluated the effects of Tai Chi on people with MDD. The results showed that exercise and yoga as adjunctive treatments could mildly reduce depression levels compared to controls, while Tai Chi lacked sufficient evidence to support its effect (Seshadri et al., 2020). Juyoung et al. evaluated the effects of yoga, tai chi, and qigong on depression, anxiety, and psychological well-being in patients with MDD. They found that MBT interventions resulted in positive outcomes, including reductions in physical pain and psychological distress, as well as improvements in physical functioning (Park et al., 2020). To validate this claim, Saeed et al. conducted a study on older adults living in nursing homes. They compared the effects of a Tai Chi intervention with usual care on the depression levels of the participants. The study found that the Tai Chi intervention group showed a significant improvement in depression levels after 12 and 24 weeks compared to the usual care group (Saeed et al., 2010). To compare the time-lapse study of MBT in antidepressant effects, Zhang et al. conducted a systematic evaluation of the effects of tai chi and qigong exercises on depression and anxiety levels of MDD patients. The study found that all subjects experienced a sustained decrease in depression and anxiety levels over the course of 57 weeks, from week 1 to week 57(Zhang et al., 2022). Effective coping strategies are necessary for individuals in special populations and those experiencing stress in modern work life. Maddux conducted a 16-week yoga intervention for employees with high levels of work-related stress and found that after 8 weeks, the yoga group showed a significant reduction in stress and negativity and a significant increase in well-being, while the blank control group showed no significant change. To re-examine the efficacy of MBT, Maddux also administered yoga to a blank control group for an additional 8 weeks and found at week 16 that the original control group also showed significant reductions in depression, stress, anxiety, and insomnia after 8 weeks of yoga (Maddux et al., 2018). Furthermore, various studies have shown the significant antidepressant effects of mind–body training in separate experiments with female college students, drug addicts, and chronic pain sufferers, among others (Liu et al., 2020; Wen et al., 2022).

From a neural mechanism perspective, studies have consistently shown that MBT has a modulatory effect on brain environment and neurotransmitters in patients with MDD. Like VPA, MBT reduces sympathetic nervous system excitability and increases parasympathetic nervous system activity, resulting in reduced stress and anxiety (Kandola et al., 2019). In terms of neurotransmitter release, several studies have demonstrated that yoga for at least 12 weeks effectively improves imbalances in patients’ positive serotonin levels and results in significant increases in levels of DA, 5-HT, BDNF and GABA (You and Ogawa, 2020; Zhang et al., 2021). In addition, MBT increases the production of endorphins in the brain and spinal cord, enhances neuroplasticity to improve the ability of neurons to form new connections with each other and adapt to change (Wang et al., 2017). Some studies have found that MBT may modulate biological processes related to depression, such as inflammation, oxidative stress, and the endocrine system. Dilorom et al. found significant reductions in IL-6, TNF-alpha, and CRP levels through systematic analyses of yoga for at least 2 weeks (Djalilova et al., 2019). Di et al. conducted a study that validated similar results. They found that 12 weeks of qigong practice increased hippocampal volume and decreased peripheral IL-6 levels compared to stretching exercises. As a result, reaction speed and sustained attention improved (Qi et al., 2021). Although these studies did not directly measure inflammatory factor concentrations in the brain, it is believed that changes in peripheral inflammation may also indicate changes in CNS inflammation. However, further research is needed to confirm this. Additionally, there is evidence that MBT may improve brain structure. Kong et al. found that regular practice of physical and mental exercise increased the volume of the hippocampus in patients with depression (Kong, 2021). In a previous study, Tao et al. discovered that 12 weeks of Ba Duan Jin and Tai Chi Chuan significantly increased gray matter volume in the insula, medial temporal lobe, and nucleus accumbens in older subjects. This was accompanied by improved memory and cognitive functioning (Tao et al., 2017). Meanwhile, MBT has been shown to activate brain regions associated with MDD patients, promote behavioral adaptive changes, and maintain the integrity of white matter volume, thereby improving brain neural processing and delaying cognitive deterioration (Zhao et al., 2020). Additionally, it modulates brain neural activity and functional connectivity in various regions, including PFC, the hippocampus/medial temporal lobe, the lateral temporal lobe, the insula, and the cingulate gyrus, as well as in brain networks such as the cognitive control network and the default mode network. Not only that, MBT improves cerebral blood flow (CBF) abnormalities in MDD patients (Liu et al., 2022), which may account for its beneficial effects on cognitive functions such as mood, attention, memory, and self-awareness (Zhang et al., 2021).

### Dose characterization of exercise for improving executive function in MDD

Higher cognitive functioning refers to the ability of humans to process information, make decisions, and control behavior in complex environments. It enables thinking, learning, memory, attention, and judgment, among other cognitive activities (Liu et al., 2022). These functions are primarily mediated by PFC of the brain. They reflect the characteristics and states of objective things and their interconnectedness, revealing their meaning and role to people (Tyng et al., 2017). Higher cognitive functions are significant and have a great influence on individuals. They aid in adapting to different situations, improving learning and work efficiency, enhancing self-regulation and social interaction, and improving the quality of life (Xiong et al., 2020). However, patients with MDD exhibit multifaceted abnormalities in higher cognitive functions, primarily manifested as executive function degradation (Rock et al., 2014). These cognitive impairments can impact patients’ daily life, social functioning, and treatment outcomes (Bernhardt et al., 2021).

#### Inhibitory control

Executive functions refer to a set of higher cognitive processes that allow individuals to intentionally regulate their thoughts, behaviors, and emotions in order to adapt to complex and changing environments (Diamond, 2013). These processes include inhibitory function, working memory, and cognitive flexibility.

Research has demonstrated that exercise can improve response inhibition and promote adaptive behavioral changes in individuals with MDD (Ahn and Kim, 2023). However, the effectiveness of exercise may be influenced by factors such as intensity, frequency, duration, and type of exercise (Greer et al., 2015). In a 6-week randomized controlled trial, Imboden divided 52 patients with MDD into two groups and administered ECT and MBT, respectively, and found that at the end of 6 weeks, the Go-No-Go task scores of the MBT group stabilized at around 393.9, while the scores of the ECT group increased from 405.5 to 443.9, which was a significant difference between the two groups. This also suggests that ECT may have a more significant effect on improving inhibitory control in MDD patients (Imboden et al., 2020).

In addition to the short-term study, Olson extended the PA intervention cycle and again found similar results. They discovered that an 8-week MICT intervention improved information processing accuracy by 1.4% in MDD patients, while the control group experienced a 2% decrease. Additionally, the exercise group experienced a decrease in reaction time by 73.45 ms, while the control group experienced an increase of 13.76 ms (Olson et al., 2017). Not only that, Alderman evaluated the effect of MBT combined with an ECT regimen on inhibitory function in 52 patients with MDD in an 8-week longitudinal study. The results showed that after the patients’ intervention, the correct rate of congruent trials increased from 96.8% at baseline to 98.5%, and the reaction time decreased from 319.5 ms to 301.2 ms. At the same time, the correct rate of incongruent trials increased from 86.1% at baseline to 89.2%, and the reaction time decreased from 390.0 ms to 373.0 ms, and all indicators showed statistically significant differences. In addition, depression and negative rumination were significantly suppressed in all patients (Alderman et al., 2016). Brüchle et al. conducted an 18-month experiment and found that the STROOP test, Go-No Go, and Tower of London scores of MDD patients significantly improved at the end of the training. This implies that long-term physical activity significantly improved the executive and inhibitory functioning of the patients (Brüchle et al., 2021).

Although PA has been shown to enhance multiple dimensions of executive functioning in patients with MDD, it is important to note that this may be due to differences in the cognitive testing tasks used in various experiments. Nevertheless, it is worth acknowledging that PA has been widely demonstrated to be an effective means of combating cognitive decline in patients with MDD.

#### Working memory

Individuals with MDD often report declines in working memory and attention, as evidenced by difficulties in memory encoding and thought retrieval, particularly the inability to retain and manipulate a limited amount of information for short periods of time (Semkovska et al., 2019).

PA has been demonstrated to be an effective method of mitigating memory and attention decline induced by MDD, but the current evidence is inconclusive. Buschert compared the effects of ECT with tetracyclic antidepressants on executive function in 38 patients with MDD in a short-term (4 weeks) randomized controlled trial. The results showed that at the end of the intervention, scores on the N-Back task, short-term verbal memory, and reaction time showed a significant upward trend in both groups compared to baseline. However, the differences between the two groups were not statistically significant. This also suggests that short-term moderate PA improves patients’ working memory more effectively than pharmacological intervention, but with fewer side effects (Buschert et al., 2019). Zhang and Chen (2019) conducted an 8-week ECT intervention and observed improvements in memory and attention among 125 patients with MDD. The Wechsler Memory Scale (WMS) results at weeks 4 and 8 showed moderate to substantial improvements in memory and attention, respectively (Zhang and Chen, 2019).

In addition to short-term studies, Brüchle found a temporary positive effect of ECT on working memory in MDD patients in an 18-month long-term intervention study. After 4 weeks, the patients showed an increase in reaction time in the 2-Back versus cross-modal matching task, but no significant change in accuracy. At week 8, accuracy on both tasks improved from 48.96% vs. 52.89 to 53.35% vs. 54.78%, respectively. However, there was no significant change in BDI-based depression scores. In the period up to the end of the experiment, the patients’ depression levels continued to decrease, but the scores on the two memory tasks did not change significantly. This suggests that in the short term, PA has a more significant improvement effect on memory function and attention than antidepressant effects in patients with MDD (Brüchle et al., 2021). In contrast, Katharina et al.’s experiment overwhelmingly refuted this claim. They found that patients with MDD did not show significant changes in attention and alertness based on the D2 Test of Attention after 8 weeks of indoor rock-climbing, despite a reduction in depression levels (Luttenberger et al., 2015). Another trial with a longer duration came to similar conclusions. Benson et al. compared 202 middle-aged and elderly MDD patients after 16 weeks of exercise or medication. It was found that the patients’ executive function improved, but there was no significant change in working memory perception on the Animal Naming Tes.

In conclusion, there is currently insufficient evidence to support the benefits of physical activity on memory and attention in MDD, but there is still much room for further exploration.

#### Cognitive flexibility

Cognitive flexibility is a crucial aspect of an individual’s judgment and thinking (Friedman et al., 2018), and it is negatively affected by the loss of BDNF due to the buildup of neuroinflammatory substances in patients with MDD. This can result in a decline in their cognitive flexibility abilities, including vision and language skills (Lima Giacobbo et al., 2019).

Although PA has been shown to improve the condition, its effects may vary depending on the type of the exercise (Morales et al., 2024). To test this claim, Viola compared the difference between the effects of ECT and MBT on cognitive flexibility in people with MDD in a 4-week longitudinal study. The results showed that the ECT group had higher scores than the MBT group on the Task Switching and Exogenous Cueing paradigms at the end of the intervention, but there was no major difference in HAMD scores between the two groups (Oertel-Knöchel et al., 2014). Similar results were found in a longer-term study in which Krogh extended the PA period to 3 months and randomized 56 patients with MDD into two groups, ECT and MBT, and found that at the end of the intervention, the ECT group’s accuracy rates on the Rey Complex Figure Test and the Digit Symbol Test for assessing visuospatial cognition increased from 56.3 and 89.5% to 61 and 93.7%, respectively, which was 1.2 and 4.7% higher than that of the MBT group. However, there was no significant difference in the BDI scores (Krogh et al., 2012). These results suggest that ECT and MBT have similar effects on improving depressive symptoms in MDD patients, but the facilitating effect on cognitive flexibility is more significant.

However, the results described above are also heterogeneous in a subset of studies. Some of the evidence suggests that ECT and MBT are similarly effective in improving MDD, while RST and EIT may be more effective than the former. Krogh, in another prospective randomized controlled trial, divided 165 patients with MDD into three groups and administered 4 months of ECT, RST, and MBT and compared the effects of the different PAs on the patients’ levels of depression and cognitive flexibility. It was found that during the first 8 weeks, HAMD scores decreased in all three groups, but there was no significant difference in the accuracy of cognitive tasks. After 12 weeks, the accuracy of the Wisconsin Card Sorting Test (WCST) in the RST group became progressively higher than in the other two groups. At the end of 4 months, the Buschke Selective Reminding Test, WCST accuracy in the RST group was already significantly higher than that of the other two groups. The HAMD scores were similar to those of the ECT and were higher than those of the MBT (Krogh et al., 2009). In addition, Brondino compared the effects of ECT and EIT on cognitive flexibility such as processing speed, similarity recognition, verbal fluency, auditory transitions and learning, complex spatial localization, and logical reasoning in a meta-analysis of 637 patients with MDD. The effect of EIT on improving cognitive flexibility was examined. The results showed that both EIT and ECT had similar effects on processing speed, verbal fluency, and auditory transitions and learning, but EIT was more effective than ECT in improving similarity recognition, complex spatial localization, and logical reasoning after more than 10 weeks of treatment, and the heterogeneity of the results was less (Brondino et al., 2017).

In conclusion, the available research suggests that there is a significant positive effect of PA on cognitive flexibility in patients with MDD, but this may be influenced by differences in the type of exercise. Most of the current evidence suggests that EIT with RST is more effective than ECT and MBT, and the present study speculates that the main reason may be due to the fact that the intensity of the exercise is higher in the former than in the latter. Nevertheless, more evidence is needed in the future to confirm the dosage profile of this PA in improving cognitive flexibility in patients with MDD.

### Correlation of exercise patterns with sleep improvement in MDD

Sleep behavior is a fundamental physiological need of the human body. MDD can significantly impact human sleep, creating a complex and bidirectional phenomenon. Individuals with MDD may experience symptoms such as difficulty falling asleep, frequent night awakenings, or early morning awakenings, which can increase the likelihood of sleep disorders and worsen depressive symptoms. This creates a vicious cycle that is difficult to break. These problems are commonly observed in patients with MDD. Firstly, there are abnormal changes in sleep structure, including a decrease in the amount of deep (slow-wave) sleep and an increase in the amount of light rapid eyes movement sleep (REM)(Vahia, 2013). Secondly, abnormal sleep states are manifested in the phenomena of difficulty in falling asleep, excessive drowsiness, waking up prematurely, or an increase in the number of awakenings, leading to fragmented sleep (Zhang, 2017). Lastly, nightmares occur frequently. This condition not only disrupts sleep quality but also causes further psychological distress. Patients may experience more frequent and vivid nightmares during symptom exacerbation or treatment (Savory et al., 2010).

PA may improve the quality of sleep in patients with MDD. However, the effectiveness of PA may vary depending on the duration of exercise, particularly on various sleep metrics. Gavin et al. investigated the immediate effect of acute ECT (>80% HRmax) on sleep efficiency in patients with MDD. They compared the effects of 30 min of ECT and sedentary reading on patients’ polysomnography-based sleep efficiency, subjective sleep quality, daytime somnolence index, and other factors. It was determined that a single session of ECT did not result in significant differences between the two groups in terms of objective or subjective sleep outcomes, daytime sleepiness, or adverse events. However, the exercise group had a positive effect on the patients’ mood states, including depression (Brupbacher et al., 2021a). In another follow-up study, this individual re-validated the non-significant effect of ECT on objective or subjective sleep indices, such as the number of awakenings during sleep and nocturnal heart rate variability, in patients with MDD (Brupbacher et al., 2021b). Although a single exercise session did not significantly improve depression, multiple studies have shown that regular physical activity can have a therapeutic effect on sleep in patients with depression. Haitham observed changes in the sleep of 82 patients with MDD by administering a HIIT intervention for 8 weeks. After training, all patients had significant reductions in their BDI and PSQI scores decreased significantly (Jahrami et al., 2022).

Additionally, exercise intensity is an important factor in enhancing sleep improvement in MDD. Markus found that elevated exercise intensity was associated with lower depressive symptoms and was accompanied by more significant improvements in mental health and sleep quality in 63 patients with MDD after 12 weeks of ECT and SIT. Simultaneously, higher exercise intensity led to a significant reduction in depressive symptoms and improved perceived health. However, further research is necessary to determine the most time-efficient, effective, and acceptable exercises for psychiatric patients in clinical settings (Gerber et al., 2019). Currently, studies have summarized the effectiveness of PA on sleep improvement in patients with MDD. Oscar found that PA intervention significantly improved the sleep quality of all patients based on both subjective and objective evaluations (Lederman et al., 2019). Meanwhile, Gavin evaluated the effectiveness of exercises, including ECT, RST, and MBT, on improving the sleep quality of patients MDD through two meta-analyses. The results showed that all exercises had a positive effect on the sleep of MDD patients. They found that MBT combined with conventional therapy and RST was significantly more effective than conventional therapy (Brupbacher et al., 2019, 2021a). On the other hand, exercise intensity is also one of the most important factors affecting an individual’s sleep quality, and Kredlow found in a meta-analysis of 2,863 subjects that acute VPA, including EIT and high-intensity RST, had superior effects on total sleep time, number of awakenings, and sleep efficiency in a single session compared with MBT and ECT. However, these differences diminished after prolonged practice of more than 1 year. Nevertheless, shorter sleep latency, stage 1 sleep, and REM sleep, as well as higher frequency of δ- and θ-band oscillation, were still observed in the long-term VPA population than in the long-term ECT vs. MBT population, suggesting that the long-term VPA population has a higher quality of deep sleep (Kredlow et al., 2015).

The effects mentioned above can be explained by neural mechanisms. This is because exercise has the ability to modulate various neurobiological factors related to depression, such as neurotransmitters, neurotrophic factors, the neurosecretory system, the neuroinflammatory response, and oxidative stress agitation. Numerous studies have demonstrated the modulatory effects of exercise on neurotransmitters such as endorphins, 5-HT, and BDNF. These neurotransmitters are associated with the regulation of mood and sleep cycles (Zimmer et al., 2016; Banno et al., 2018). Additionally, exercise increases the availability of tryptophan in the brain, which promotes better sleep quality by increasing 5-HT levels. Exercise has been found to modulate the NE system and release NE, a system and transmitter involved in regulating arousal, attention, and stress sensing. This is important for individuals to stay awake and focused during the day, while promoting a more restful sleep process at night (Leuenberger et al., 1993). Meanwhile, PA can also reduce the stress response of patients with MDD by influencing neurosecretory systems, such as the hypothalamic–pituitary–adrenal axis (HPA axis) and hypothalamic–pituitary-gonadal axis (HPG axis), and lowering the levels of hormones, such as cortisol and estrogen (Ignácio et al., 2019). In addition, PA increases the activity of antioxidant enzymes, such as glutathione peroxidases (GPx) and superoxide dismutase (SOD). This reduces neuroinflammatory reactions and oxidative damage caused by free radicals to brain tissues, thus protecting the normal function of sleep nerves (Elokda and Nielsen, 2007).

## Possible mechanisms by which PA improves MDD symptoms

### PA improves MDD by remodeling brain structure

The impact of PA on brain structure is a broad area of research. Numerous studies have confirmed that PA has positive effects on brain structure, such as promoting neurogenesis and neural regeneration, increasing gray matter volume, improving white matter fibers, increasing CBF, and enhancing cognitive function (Burdette et al., 2010; Erickson et al., 2012). However, in patients with MDD, some of the significant causes of symptoms, such as cognitive decline, slow thinking, and sleep abnormalities, are believed to be associated with abnormal changes in brain structure (Frodl et al., 2008). These changes include hippocampal atrophy, thinning of the PFC, abnormalities in amygdala volume, decreased motor cortex thickness, and neuronal inactivation in dense areas of the substantia nigra (Mikkelsen et al., 2017).

#### Neuroconnectivity of the basal ganglia

For intracerebral structures, exercise has been shown to promote neurogenesis and neuroplasticity in the brains of patients with MDD, improving cognitive function and emotion regulation, as well as contributing to the balance of the autonomic nervous system. Additionally, it increases the volume of gray and white matter in the cerebrum and cerebellum, specifically for intracerebral structures. Research has shown that PA can increase neurotransmitter content in the mesencephalon and promote neuron generation and regeneration in the brainstem, leading to improved brainstem function. This improvement can help MDD patients regulate their moods and improve sleep quality. Additionally, a study found that depressed patients had a 6.4% reduction in cerebellar gray matter volume compared to healthy controls. Regular exercise may increase cerebellar gray matter volume, which could potentially help alleviate symptoms such as depressed mood (Gujral et al., 2017). A recent study utilized multimodal neuroimaging techniques, such as diffusion, anatomical, and quantitative MRI, to investigate the microarchitecture of mesencephalic and subcortical regions in depressed patients. The study found that these patients had deficits in these regions, including the shell nucleus and thalamus. Subjects with disrupted structural connectivity within and between the caudate nucleus and thalamocortical network, who underwent exercise interventions, showed improved mesencephalic microarchitecture compared to a blank control group. This improvement was associated with better cognitive functioning (Zhang et al., 2022).

#### Prefrontal cortex density

Recent innovations in neuroimaging techniques have led to increased interest in PA-induced changes in PFC and related cognitive networks. Studies have shown that patients with MDD may experience a decline in executive functions due to decreased density and volume of PFC, anterior cingulate cortex (ACC), orbitofrontal cortex (OFC), dorsolateral PFC (dlPFC), and dorsomedial PFC (dmPFC), while exercise may increase their size and function (Van Dam and Chrysikou, 2021; Pizzagalli and Roberts, 2022)。However, severial studies have found a positive correlation between the level of PA and the volume of the PFC (Gordon et al., 2008; Bugg and Head, 2011). One study found that moderate ECT mitigated age-related atrophy of the frontal, parietal, and temporal cortices, and improved the density relationship of tissues within the brain as observed by MRI (Colcombe et al., 2003). Two additional studies with long-term follow-up also reached similar conclusions. They found a strong association between subjects’ PFC volume in later life and their PA levels in midlife. Additionally, after WML rating by FLAIR images, individuals with exercise habits showed higher total brain volume, gray matter volume, and density in frontal regions in later life (Rovio et al., 2010). Another study reported a correlation between regular PA and ACC volume changes. Two six-month studies found that MPA, including walking, increased the volumes of subjects’ ACC and PFC. However, this effect may be attenuated by age. For example, the elderly population was noted to require at least six months of physical activity to produce a similar effect (Ruscheweyh et al., 2011). This is considered to be one of the important mechanistic reasons why physical activity can improve executive dysfunction in patients with MDD compared to older MDD populations.

#### White matter integrity

In addition to gray matter, white matter and the corpus callosum are important components of the brain’s structure. They consist of nerve fibers that transmit electrical signals between different regions of the brain. Several studies have demonstrated that PA can have a positive effect on white matter, enhancing its structural integrity and function. For instance, Erickson et al. found that ECT resulted in increased white matter integrity in several brain regions, including the corpus callosum, and improved connectivity between the two hemispheres of the brain. This, in turn, led to improvements in subjects’ memory function (Erickson et al., 2011). This study demonstrated the beneficial effect of ECT on brain and cognitive health in children. Laura et al. used diffusion tensor imaging (DTI) to compare the brain health of physically fit children to their less fit peers. The results showed that the physically fit children had better anisotropy in the corpus callosum, corona radioulnaris, and portions of the superior longitudinal fasciculus, as well as higher white matter integrity in several brain regions (Chaddock-Heyman et al., 2014). Meanwhile, Michelle found in a year-long study that walking exercise significantly improved white matter integrity, short-term memory, and executive function in older adults (Voss et al., 2013). Furthermore, additional studies have examined the impact of various forms of PA on white matter integrity. Ludyga et al. discovered that HIIT over a short period of time (3 weeks) led to noteworthy enhancements in white matter integrity, as well as more substantial improvements in verbal memory (Zimmer et al., 2018).

#### Striatum volume

Furthermore, numerous studies have confirmed the positive effects of PA on the striatum, hippocampus, and amygdala in individuals with MDD compared to those without. The striatum’s involvement in reward processing and motivation has also been linked to depression. Various studies have reported a decrease in striatal volume in individuals with MDD (Koolschijn et al., 2009; Bora et al., 2012). Although the number of studies in this area is limited, there is evidence that PA may have a positive effect on the striatum in patients with MDD. Krogh et al. found that acute ECT can lead to increased levels of activation in the striatum in patients with MDD when compared to blank controls (Krogh et al., 2014). Timothy found a positive correlation between PA and the dorsal striatum volume in older adults, as well as an increase in the volume of the ventral nucleus ambiguous (Verstynen et al., 2012). However, a study in older adults demonstrated that although regular exercise increased the hippocampal volume of the subjects, it did not have a significant effect on the volume of the caudate nucleus (Kandola et al., 2016). A meta-analysis revealed that LLD is associated with impaired structural integrity of the frontal-limbic network, including the amygdala (Wen et al., 2014). These studies all suggest that PA may enhance neurogenesis and synaptic plasticity in these regions, and may be associated with striatal volume, potentially affecting depressive symptoms.

#### Hippocampal plasticity

Another part of the research has focused specifically on hippocampal plasticity. Erickson et al. found in a series of studies that higher levels of corticotropin-releasing factor were associated with greater hippocampal volume (Erickson et al., 2009). For instance, ECT has been found to be effective in enhancing hippocampal function and promoting neuroplasticity (Kandola et al., 2016). In a previous study, the author assessed hippocampal volume in 165 older adults on a spatial memory task using the Maximum Graded Motor Test and f-MRI. The study found that higher task scores were associated with larger bilateral hippocampal volumes (Erickson et al., 2010). In a second 12-week study, the author found that regular ECT was associated with changes in cerebral blood volume, dentate gyrus, and cardiorespiratory health in adults (Pereira et al., 2007). Another study of 120 older adults found that the exercise group had a 2% increase in hippocampal volume, while the control group showed no changes in brain structure or cognitive performance (Erickson et al., 2011). This confirms the link between PA levels and hippocampal volume, which has a positive effect on memory performance (Erickson et al., 2011). This indicates that exercise may improve negative mood through its effects on the amygdala and the limbic network’s interaction, modulation, and control of cognitive functions. A study demonstrated that the exercise group had a significant increase in the volume of the hippocampal CA1 and DG regions after 4 weeks of running compared to the control group (Chen et al., 2017). Additionally, studies have examined the impact of various types of exercise on the hippocampus. Niemann et al. discovered that both ECT and stretch and relaxation training resulted in an increase in hippocampal volume in elderly subjects, as determined by MRI after 12 months. However, the type of exercise did not appear to affect this outcome (Niemann et al., 2014).

### PA improves intracerebral microenvironment to alleviate MDD

MDD can have a wide range of effects on the brain’s intracerebral environment. Studies have shown that MDD is associated with a decrease in 5-HT, NE (Kessing et al., 2024), DA, CBF (Wiciński et al., 2020), and changes in cellular growth factors and OS markers, which can lead to neuronal damage and worsen MDD (Chen et al., 2024). However, several studies have also shown that PA has a positive impact on improving the brain’s intracerebral environment in individuals with MDD. For instance, it can increase levels of 5-HT and DA (Smith et al., 2019), modulate ion release and metabotropic receptor signaling pathways, affect neuronal excitability and synaptic plasticity (Dai et al., 2024), and improve small molecules such as Glu, GABA, BDNF, and cortisol (Drevets et al., 2008; Felger and Lotrich, 2013). However, the relationship between PA levels and depression is uncertain in terms of dose–response and may depend on various factors, such as the type, intensity, frequency, and duration of the activity.

#### Regulation of cerebral blood flow

Regarding CBF, PA enhances cerebrovascular function, oxygen and glucose delivery, neuronal activity, and neurovascular coupling (Querido and Sheel, 2007). Alfonso et al. investigated changes in CBF before and after a 12-week aerobic exercise intervention in the resting state. The study found that the exercise group had increased CBF in the frontal cortex and hippocampal regions (Alfini et al., 2019). Sandra et al. conducted a 6-week ECT study on older adults’ CBF. The results showed an increase in CBF in the anterior cingulate gyrus region in the exercise group (Chapman et al., 2013). In contrast, people with MDD had significantly lower cerebral perfusion in the hippocampal region. Although exercise may help improve cerebrovascular function in depressed patients (Chen et al., 2017), the underlying mechanisms of this effect have not been fully elucidated. Chen et al. conducted two animal experiments to compare the effects of regular running on various parameters of capillaries in the brain of a depressed rat model. However, it is important to note that sucrose consumption was significantly increased in the exercise group. It is possible that this increase in sucrose consumption may have affected the results. The results showed that regular running had a positive effect on total capillary volume, total length, and total surface area in the CA1 area and dentate gyrus of the rat hippocampus. Meanwhile, the dentate gyrus may be a structural basis for exercise-induced treatment of MDD (Chen et al., 2016, 2017). Additionally, acute MICT has been shown to increase resting cerebral blood flow and cerebral metabolism via glucose, which may contribute to the improvement of neuropsychiatric symptoms caused by hypoperfusion or hypometabolism (JN, 2015). This suggests that PA may have a positive impact on improving cerebral blood flow in patients with MDD.

#### Repair of nerve cells

Glial cells regulate the strength of synaptic connections between nerve cells, and changes in intracellular calcium may play a key role in this process (Malchow et al., 2021). In an animal model of MDD, glial loss was observed in prefrontal and limbic brain regions. Degeneration of astrocytes leads to glutamate excess in the synaptic cleft and glutamate/GABA imbalance in other affected structures (Śmiałowska et al., 2013). Furthermore, patients with MDD exhibit a significant reduction in the density and size of neurons and glial cells in both the ependymal and hypodermal cortex of the dorsolateral PFC (Rajkowska et al., 1999). Additionally, PA has been found to potentially have a beneficial effect on single neurons, glia, nerve fibers, and synapses. Exercise has been shown to improve the function and morphology of glial cells, including astrocytes, oligodendrocytes, and microglia. These cells support neuronal metabolism, myelination, immune responses, and synaptic transmission (Mee-Inta et al., 2019). Lisiani et al. discovered that PA increased the morphology, density, and expression of glial fibrillary acidic protein (GFAP) in astrocytes from the CA1 region of the rat hippocampus through animal experiments (Saur et al., 2014). Similarly, Marina found that early PA improved astrocyte-microcirculation communication and reduced microglia activation in a rat model of chronic cerebral underperfusion (Leardini-Tristão et al., 2020). In observational studies on human subjects, Kaitlin found that older adults who maintain exercise over a long period of time have stronger neurotransmission in the brain. This promotes the secretion of brain chemicals that protect aging synapses. Additionally, exercise increases levels of presynaptic proteins, which are involved in synaptic function and plasticity. These findings demonstrate that PA levels are related to synaptic integrity (Casaletto et al., 2022).

Additionally, PA has been shown to have a positive impact on certain neurons in patients with MDD. Specifically, exercise activates GAD2 neurons located in the substantia nigra pars reticulata. These neurons not only connect to brain regions responsible for motor control, but also project to areas that regulate brain states, such as the dorsal nucleus of the dorsal raphe (DR), the nucleus of the locus coeruleus (LC), and the ventral tegmental area. These neurons can inhibit motor brain regions and the brain’s wakefulness centers, terminating movement and initiating sleep and other behaviors (Wu et al., 2011). Exercise has been found to increase activity in the nucleus accumben (NAc), which is involved in rewarding behaviors and consists of two types of medium spiny neurons (MSNs) expressing DA receptors D1 or D2. These MSNs mediate the outcome of failure or stress associated with depression. Moderating them can improve mood and motivation in depressed patients (Francis et al., 2015). For instance, Zhuang and colleagues investigated the impact of treadmill exercise on depressive behaviors, as well as spine density and morphology of major neurons in rats. The study focused on the hippocampus, medial prefrontal cortex (mPFC), NAc, and basal amygdala (BLA) affected by chronic unpredictable stress. The study found that exercise reduced depression-like behaviors and restored spine density and morphology in specific brain regions (Zhuang et al., 2019). Feng et al. also utilized treadmill exercise and reported the effects on protein expression related to the hippocampus, dendritic morphology of the cerebral cortex, and synaptic plasticity in a mouse model of depression induced by ovariectomy followed by menopause. The study demonstrated that exercise had a positive effect on dendritic morphology and increased the expression of BDNF and mammalian target of rapamycin (mTOR) signaling pathway proteins in multiple brain regions (Feng et al., 2021).

#### Neurotransmitter facilitation

The treatment of MDD with PA can be explained by various factors, including neurotransmitters, small molecules, and ion channels. Existing animal studies and genetic associations suggest that certain channels, such as transient calcium channel (T), neuronal calcium channel (N), voltage-gated potassium (Kv), 5-hydroxytryptamine type 3 (5-HT3), and ligand gated ion channel 7 (P2RX7), may influence depressive-like behaviors. Additionally, exercise may affect the expression and function of voltage-gated sodium channels Nav1.3, Nav1.7, and Nav1.8, which are involved in neuronal excitability and synaptic transmission (Lodge and Li, 2008). It has been suggested that exercise can affect the activity of GABA-A receptors, which are chloride channels that mediate inhibitory neurotransmission. Additionally, exercise may alter the levels of calcium-binding proteins, which help maintain calcium ion balance and protect neurons from oxidative stress and apoptosis. These physiological changes are similar to those seen with antidepressant medications (Kandola et al., 2019). Other studies have also found that PA can affect the signaling pathways of metabolic receptors, such as G-protein coupled receptors (GPCRs), which may improve synaptic transmission and neurogenesis (Blad et al., 2012; Liu et al., 2021; Yang et al., 2021).

Furthermore, recent research has concentrated on the molecular foundation of exercise for enhancing mood. PA has been shown to have a positive impact on mood states through various physiological and biochemical mechanisms, such as endorphins, mitochondria, mTOR proteins, neurotransmitters, and the hypothalamic–pituitary–adrenal axis, as well as the caloric hypothesis (Mikkelsen et al., 2017). For instance, 5-HT, a neurotransmitter that regulates various neuropsychiatric processes, has been linked to the development of depression. Research has demonstrated that individuals with endogenous MDD exhibit reduced levels of plasma tryptophan, which is a precursor to serotonin (Hong et al., 2022). Numerous studies on both humans and animals have shown that extended physical activity boosts the synthesis and release of serotonin, enhances the expression and function of 5-HT receptors, and reduces 5-HT degradation and reuptake, thereby prolonging its availability and action in the synaptic cleft (Melancon et al., 2014; Yuan et al., 2015; Cordeiro et al., 2017). As a result, 5-HT regulates neuronal activity and plasticity, making it one of the key mechanisms by which exercise enhances mood (Franco et al., 2021).

Another neurotransmitter associated with reward, motivation, and learning is DA. Individuals with MDD often exhibit dysfunction in the DAergic system, resulting in a lack of interest and pleasure (Belujon and Grace, 2017). On the other hand, PA has been shown to stimulate the activity of DAergic neurons and increase the concentration of DA in the synaptic gap, thereby improving mindfulness and cognitive function (Kip and Parr-Brownlie, 2023). Exercise leads to elevated serum calcium levels, which allows calcium to be transported to the brain and enhance dopamine synthesis via a calmodulin-dependent system. Elevated dopamine levels regulate various brain functions, such as mood, reward, learning, and memory (Sutoo and Akiyama, 2003). Simultaneously, PA-promoted DA synthesis increased synaptic adaptation to repeated stimuli, leading to an increase in hippocampal neurogenesis. These changes may be related to alterations in the DA system (Van Praag, 2008).

Alternatively, PA may be associated with the production and increase of neurogenic factors such as BDNF, vascular endothelial growth factor (VEGF), insulin-like growth factor 1 (IGF-1), fibroblast growth factor 2 (FGF-2), and endogenous opioid peptides, which may promote neuronal survival, differentiation, maturation, and integration (Vonderwalde and Kovacs-Litman, 2018; Qi et al., 2022). Sartori’s animal studies have shown that PA exercise increases BDNF expression in the hippocampus and blood vessels, promotes VEGF circulation, and improves the hypothalamic–pituitary–adrenal (HPA) system. Autonomously exercising rats also showed robust increases in mature BDNF protein levels as well as p11 (a gene associated with BDNF precursor cleavage), tissue plasminogen activator (tPA), and hippocampal mRNA expression compared to sedentary rats (Sartori et al., 2011). Zheng et al. similarly verified the BDNF-stimulating effect of exercise through experiments based on a rat model of unpredictable stress-induced depression (CNS) and also found that exercise counteracted the CNS-induced reduction of BDNF and mRNA in the hippocampus of rats (Zheng et al., 2006). Morland et al. investigated the delayed neurodegenerative effects of VPA and found that exercise increased VEGF protein and capillary density in mice after a 7-week HIIT intervention (Morland et al., 2017). Other studies in animal models have similarly shown that exercise promotes higher levels of BDNF in the MDD and normal brain, while possibly additionally reducing the risk of PD or AD (Voss et al., 2013). It has also been discussed that exercise triggers several other cellular and molecular changes that contribute to neuroplasticity, such as the release of insulin growth factor-1 (IGF-1), fibroblast growth factor (FGF), β-endorphin, and lipocalin (Bolijn and Lucassen, 2015).

It has also been reported that long-term ECT not only promotes the release, transport, and metabolism of Glu, γ-aminobutyric acid, GABA, NE (Duman et al., 2019; Morres et al., 2019), and other monoamines in the hippocampus/central/medial amygdala in patients with MDD, but also increases the degree of sympathetic nervous system activation, which increases the ability to synthesize and release NE^212,213^. Dunn, for example, found in animal models that both long-term treadmill and autonomic running increased NE levels in the pontine medulla and spinal cord compared to sedentary controls (DUNN et al., 1996). And studies have shown that a single session of ECT can promote endogenous NE activity, thereby enhancing memory and responsiveness (Segal et al., 2012). In addition, human plasma glycophorin (hGAL) levels are significantly elevated after acute exercise. However, the stimulation of the monoamine system may depend on the intensity of the exercise, e.g., in animal studies, treadmill exercise was more effective in increasing serum corticosterone levels than autonomous wheel running (Liu et al., 2009). This is mainly due to the fact that different intensities based on the two types of exercise may induce different degrees of feedback on the hypothalamic–pituitary–adrenal axis (Lin and Kuo, 2013).

#### Suppressing inflammation

Of all the small molecule changes in the brain, PA has also been shown to reduce inflammation through several different processes (inflammation, cytokines, class I receptors, adipose tissue, and vagal tone), which may contribute to improved health outcomes in patients suffering from mood disorders (Mee-Inta et al., 2019). For example, in animal studies, PA has been shown to improve mitochondrial function in the rat cerebral cortex and cerebellum, reduce OS and apoptosis-related markers, and induce favorable changes in mitochondrial biogenesis, dynamics, and autophagy signaling, which may also lead to increased mitochondrial plasticity, resulting in a more robust phenotype (Isaacs et al., 1992; Marques-Aleixo et al., 2015). And in patients with MDD, exercise has been shown to reduce levels of pro-inflammatory cytokines such as IL-6, interleukin-10 (IL-10), TNF-α, transforming growth factor-β (TGF-β), and CRP, which may contribute to neuroinflammation, oxidative stress, and neurodegeneration (Pruimboom et al., 2015; Venkatesh et al., 2020). For example, two human studies showed that both ECT and RST reduced serum levels of IL-6, TNF-α, and CRP. However, when exercise was discontinued, plasma levels of IL-6 and CRP increased, as did perceived stress and negative emotions (Hamer et al., 2012; Venkatesh et al., 2020). In addition, Francisco et al. investigated the effects of 12 weeks of MICT on gut microbiota composition, inflammatory markers, and depressive symptoms in patients with MDD. The results showed that exercise had a modulating effect on the microbiota-gut-brain axis, e.g., increasing the abundance of beneficial bacteria such as Bifidobacteria and Lactobacillus, and improving MDD symptoms (Donoso et al., 2023). Therefore, it has been speculated that the mechanistic reason for the effect may be that plasma improves memory and reduces neuroinflammation in the hippocampus by delivering extracellular vesicles carrying anti-inflammatory microRNAs during exercise in both mice and humans, thereby reducing negative mood factors, including depression (de Miguel et al., 2021).

### Characterization of neural electrical activity during PA intervention in MDD

The cerebral cortex contains neurons that exhibit bioelectrical activity, resulting in sustained rhythmic potential changes known as spontaneous EEG activity. In recent years, the field of brain neuroscience has rapidly developed, leading to the increased use of neuroimaging techniques such as electroencephalography (EEG) and event-related potentials (ERP) in psychology (Daume et al., 2024). Among them, EEG is a graph obtained by amplifying and recording the information of spontaneous bioelectrical potentials inside the brain. It can observe the spontaneous and rhythmic electrical activities of brain cell populations. On the other hand, ERP is a special kind of brain evoked potentials that is detected by highly sensitive electrodes and amplifiers. It provides indicators such as the wave amplitude, latency, and spatial distribution of the potentials, and the spectrum of the potentials to understand the neurophysiological changes in the brain during cognitive processes. Patients with MDD may exhibit higher alpha, theta, and delta wave power and lower beta and gamma wave power in their EEG signals compared to the normal population. These changes may reflect impaired cognitive function in depressed patients (Newson and Thiagarajan, 2018; de Aguiar Neto and Rosa, 2019). Additionally, patients with MDD may also exhibit abnormalities in some ERP components, such as P50, N100, and P300, which reflect impaired sensitivity to stimuli, processing ability, and emotion regulation (Light et al., 2010).

Studies have shown that long-term PA can have beneficial effects on patients with MDD in terms of EEG characteristics and mechanisms. Using the amplitude of each band of the EEG as an outcome measure, Cooney found in a meta-analysis involving 2,326 patients with MDD that long-term regular PA increased patients’ alpha band (10 Hz-13 Hz) activity and suppressed beta (14 Hz-30 Hz) and theta (4 Hz-7 Hz) band amplitudes at rest; increased patients’ delta band (0. 5 Hz-3 Hz) activity; and elevation of patients’ midrange-β band (13 Hz-20 Hz) and high-β band (20 Hz-30 Hz) oscillations during cognition and learning. Meanwhile, the majority of these signals originated from central, prefrontal, and motor areas. This phenomenon may be one of the neural mechanisms by which PA improves depressive symptoms and cognitive function in MDD patients (Cooney et al., 2013). A study conducted by Silveira showed that depression in old age is associated with lower mean EEG frequency in the cerebral cortex. In the first study, she compared changes in geriatric depression and healthy individuals over 6 months of ECT. The results showed that patients had lower theta wave frequencies in regions P3, P4, T5, T6, O1, and O2 than healthy controls. After ECT, patients experienced an increase in these frequencies and a decrease in HAMD scores (Silveira et al., 2010). This supports previous studies that suggest exercise can promote arousal and improve depression by activating cortical activity. Additionally, Chan et al. conducted MBT on patients with MDD and found elevated levels of frontal EEG lateralization and left frontal hemisphere regions consistent with theta waves. No significant changes were observed in the other groups, indicating that LPAs such as MBT can activate positive emotions and improve attention (Chan et al., 2013).

In addition to prolonged exercise, some studies have demonstrated that acute exercise can increase alpha, beta, and delta versus theta band activity (Crabbe and Dishman, 2004). Furthermore, after incorporating various forms of PA such as walking, running, cycling, and swimming, similar activation patterns were observed in different brain regions, including the frontal, temporal, central, parietal, and occipital cortices, within 30 min after exercise. This suggests that the health benefits of exercise are less influenced by the form of PA (Mechau et al., 1998). A study was conducted to examine the effects of different exercise intensities (ECT/EIT) on depressive symptoms and subjective sleep in normal individuals using EEG. The results showed that VPA was more effective in reducing depressive symptoms and light sleep percentage, while increasing total sleep time, stage 4 sleep, REM sleep, and slow wave sleep (Gerber et al., 2014). These findings suggest that exercise intensity may be related to the reduction of MDD symptoms and improvement of mental health through EEG signaling mechanisms. It is difficult to capture electrophysiological changes in the human brain during exercise. Therefore, several animal studies have been conducted to record neuroelectric changes in rodents via microelectrodes and local field potentials (LFP). These studies have found an increase in high and medium frequencies (6.5–12 Hz) of rodent theta power through autonomous exercise. Hippocampal theta oscillations were always present, and spatial memory was enhanced in many spatial memory-based tasks (Li et al., 2008; Buzsáki and Moser, 2013; Li et al., 2021). This may be due to the fact that during acute exercise, the concentration of acetylcholine around the hippocampus increases (Buzsáki, 2005; Giocomo and Hasselmo, 2007), which enhances the frequency of internal interneuron discharges (Hasselmo and Eichenbaum, 2005), and since the prefrontal cortex is the final receiver of this cascade of activity (Li et al., 2015), the hippocampal-prefrontal interactions exhibit a sustained exercise-induced theta oscillation (Fujisawa et al., 2008).

However, studies on exercise and ERP in people with MDD have not yielded consistent results. For instance, one study compared MBT and ECT in terms of their effectiveness in improving executive ability based on ERP. The study found that ECT reduced reaction time in all subjects compared to MBT and baseline. Additionally, there was a decrease in P3 latency and an increase in P3 amplitude in all subjects (Kamijo et al., 2009). Furthermore, other studies have reported similar findings to the aforementioned study, demonstrating a comparable increase in P3 amplitude and decrease in P3 latency when performing a cognitive task after acute exercise (Chang et al., 2015, 2017). Although the specific neural mechanism of the P3 component is not yet fully understood, some studies suggest that it may be mediated by interactions between the frontal and temporal/parietal cortices, which are facilitated by norepinephrine and dopamine (Polich, 2007). This reflects the degree of synchronized firing of a large number of excitatory pyramidal neurons in the cortex (Sur and Sinha, 2009). Studies have investigated the impact of single sessions of ECT on positive emotional responses in patients with MDD through late positive potential (LPP) in ERP. The results showed that a single exercise session increased LPP in healthy individuals, but no such change was observed in patients with short- or long-term MDD. Additionally, a significant increase in LPP was observed in emotionally reactive intact individuals compared to emotionally reactive impaired individuals (Brush et al., 2021). An earlier study by Samii compared the effects of a single session of RST on motor evoked potentials (MEPs) in normal and MDD patients. The study found that all subjects experienced a boost in MEPs after exercise, but the increase in the depressed group was significantly lower than the normal level (Samii et al., 1996).

## Limitations and suggestions for future research

### Precise control of total exercise dose during each training cycle

The effects of PA on improving depressive symptoms, cognitive function, and sleep status in patients with MDD have been widely demonstrated, but different types, durations, and intensities of PA may lead to differences in the effects of interventions for MDD. Therefore, to ensure the optimal effect of PA in MDD intervention, this study suggests that the dosage characteristics of PA in different MDD populations should be emphasized before developing exercise prescriptions. Therefore, two main considerations should be made. First, the population should be divided into different sexes and ages with different degrees of MDD symptoms. For example, in terms of age screening, EIT or RST can be used regularly for young patients (Martland et al., 2020), while low-intensity ECT should be used more often for older patients due to functional limitations (Heinzel et al., 2022). In terms of population differences, positive mindfulness-based MBT can be used for patients with postpartum depression (Park et al., 2020), whereas for treatment-resistant depression, a combination of pharmacologic agents such as ECT combined with tricyclic antidepressants, tetracyclic antidepressants, and monoamine oxidase inhibitors is used (Li et al., 2023). Second, after determining the population and PA type, one should proceed to plan the total training load within each rehabilitation cycle (typically 8 weeks/2 months). During this process, the patient’s physiological and psychological feedback at different time points can be evaluated using specialized training load monitors such as TRIMP and s-RPE (Menaspà et al., 2018)。In this way, the patient’s rehabilitation progress can be understood in a timely manner and the current and future training program can be adjusted and predicted.

### Rationalize the volume of a single workout to enhance exercise efficiency

Once the exercise dose within each training cycle (8 weeks/2 months) has been determined, the exercise program for a single training session (same day) is planned. Among other things, it is important to control the level of physiological and psychological load of the single training session for individuals with MDD. This is because, unlike healthy individuals, patients with MDD generally have lower exercise compliance due to symptoms such as low interest and low motivation to exercise (Vancampfort et al., 2015). Therefore, in order to improve the effectiveness of exercise as much as possible on the basis of ensuring exercise compliance, this study concluded that two main points should be considered. First, according to the “appropriate load principle (Impellizzeri et al., 2022),” regardless of the difference in the type of PA, the intensity and load of a single training session should first be reduced to ensure the continuity and integrity of the patient’s training in a single session. At the same time, according to the “motivational principle in sports (Schmid et al., 2020),” the training difficulty should be reduced over time according to the patient’s performance during the intervention process, so as to stimulate his/her initiative to complete the training. Second, according to the “Progressive Overload Training Principle (Impellizzeri et al., 2020),” the training program should be updated regularly, and different types of exercises should be cross-arranged in each training session. The purpose of this approach is to enrich the form of PA to improve patients’ motivation and adherence to exercise, and at the same time, gradually increase the intensity and load of exercise to improve the effect of the intervention on MDD.

### Exercise combined with multimodality monitoring into the MDD healthcare system

In addition to emphasizing the intervention effect of PA on MDD patients, it should also be followed up in the existing medical system and technology of depression rehabilitation. Unlike the goal of drug intervention, PA can improve MDD in many ways. Adequate exercise can not only reduce the depression level of an individual, but also reverse the cognitive function impairment and decline in sleep quality caused by MDD. However, to date, exercise intervention has only been used as an adjunctive treatment for MDD in some developed countries and regions, and has not received the same attention as drug treatment, so its clinical value and medical recognition still need to be improved. In order to accelerate the progress of incorporating PA into the MDD healthcare system, this study suggests that hospitals and rehabilitation organizations can support it in two ways. On the one hand, the most common assessment modalities of MDD at present are behavioral observation, scale assessment, and clinician’s clinical experience. With the current advances in multimodal brain function monitoring technology, fMRI, EEG, and NIRS can be used in basic research and gradually applied to the process of patient follow-up during rehabilitation. A large number of studies have reported on the neuropathological mechanisms of psychiatric disorders such as bipolar disorder, schizophrenia, and MDD using these techniques (Abi-Dargham et al., 2023; Wolff and Northoff, 2024). Although the cost of using such techniques may be high, the Declaration of Helsinki emphasizes that physicians should prioritize the patient’s right to health. Even when the best interventions are proven, their safety and efficacy must be continually evaluated (W.M. Association, 2013). Therefore, this study suggests that the use of multimodal monitoring technology in MDD outpatient and recurrent visits could be pioneered in some regions, such as Scandinavia and the Americas, and cost containment considerations could be taken into account once the frequency and acceptance of its use has reached a certain level. On the other hand, although exercise has been used clinically as a common intervention in the prescription of metabolic syndrome and obstructive sleep apnea, the most common prescription for MDD is still medication, and exercise interventions have only appeared in most basic studies (Mangione et al., 2022; Duan et al., 2024). However, as the significant interventional effects of PA on MDD continue to be demonstrated, hospitals and rehabilitation facilities in some regions may also include exercise as an adjunctive treatment modality in their formularies and update them regularly while ensuring patient safety. Ultimately, exercise intervention can be integrated into the existing MDD healthcare system.

## Conclusion and recommendation

PA has a significant ameliorative effect on MDD, but there may be differences in the effects of different types of exercise. The available literature suggests that EIT has the most significant effect on reducing depression levels in patients, ECT has the highest frequency of use and clinical acceptability, MBT has the best adherence, and RST may have a similar or slightly smaller effect than EIT, but the literature is limited. However, the effects of combined exercise or exercise combined with pharmacological interventions may be superior. Through multimodal monitoring, it was found that the neurological mechanism may be that PA significantly improved hippocampal volume, cortical density, and regulated the release of neurotransmitters such as monoamines and neuropeptides in patients with MDD. Therefore, PA may be used as an adjunctive tool in future research and clinical treatment of MDD.

## Author contributions

JG: Writing – original draft, Writing – review & editing, Methodology, Supervision. YS: Writing – review & editing, Supervision, Conceptualization, Formal analysis. YF: Data curation, Supervision, Validation, Writing – original draft. HY: Conceptualization, Writing – original draft, Writing – review & editing. JAL: Methodology, Project administration, Writing – review & editing, Investigation. CL: Methodology, Project administration, Writing – review & editing, Data curation. JNL: Writing – review & editing, Project administration, Funding acquisition, Resources.

## Glossary

Abbreviation: Full Name
WHO: World Health Organization
ACSM: American College of Sports Medicine
MDD: Major Depressive Disorder
TRD: Treatment-Resistant Depression
PA: Physical Activity
ECT: Endurance Continuous Training
EIT: Explosive Interval Training
RST: Resistance Strength Training
MBT: Mind–Body Training
AE: Aerobics Exercise
MICT: Moderate-Intensity Continuous Training
MET: Metabolic Equivalent
VPA: Vigorous Physical Activity
HIIT: High-Intensity Interval Training
SIT: Sprint Interval Training
BRMS: Bech-Rafaelsen Melancholy Scale
CES-D: Center for Epidemiologic Studies Depression Scale
HAMD: Hamilton Depression Scale
BDI: Beck Depression Inventory
PHQ-9: Patient Health Questionnaire
Q-LES-QSF: Quality of Life Enjoyment and Satisfaction Questionnaire-Short Form
MADRS: Montgomery-Asberg Depression Rating Scale
SF-36: MOS item short from health survey
STAI: State–Trait Anxiety Inventory
PSS-10: Perceived Stress Scale
CD-RISC: Connor-Davidson Resilience Scale
QIDS: Quick Inventory of Depression Symptomology
HAMA: Hamilton Anxiety Scale
MAAS: Mindful Attention Awareness Scale
WMS: Wechsler Memory Scale
WCST: Wsiconsin Card Sorting Test
REM: Rapid Eyes Movement Sleep
DTI: Diffusion Tensor Imaging
MRI: Magnetic Resonance Imaging
fMRI: Functional Magnetic Resonance Imaging
NIRS: Near Infrared Spectrum Instrument
EEG: Electroencephalogram
ERP: Event-Related Potential
LFP: Ocal Field Potentials
LPP: Late Positive Potential
MEPs: Motor Evoked Potentials
MCA: Middle Cerebral Artery
CBF: Cerebral Blood Flow
CNS: Central Nervous System
HPA: Hypothalamic–Pituitary–Adrenal
HPA axis: Hypothalamic–Pituitary–Adrenal Axis
HPG axis: Hypothalamic–Pituitary-Gonadal Axis
PFC: Prefrontal Cortex
mPFC: Medial Prefrontal Cortex
dlPFC: Dorsolateral Prefrontal Cortex
dmPFC: Dorsomedial Prefrontal Cortex
ACC: Anterior Cingulate Cortex
SNR: Substantia Nigra Pars Reticulate
NAc: Nucleus Accumbens
MSNs: Medium Spiny Neurons
GAD2: GAD2 Neurons
DR: Dorsal Raphe
LC: Locus Coeruleus
BLA: Basal Amygdala
5-HT: 5-Hydroxy Tryptamine
NE: Norepinephrine
BDNF: Brain-Derived Neurotrophic Factor
DA: Dopamine
Glu: Glutamate
GAD2: Glutamate Decarboxylase 2
GABA: Gamma-Aminobutyric Acid
APP: Acute Phase Protein
OS: Oxidative Stress
IL-1: Interleukin-1
IL-6: Interleukin-6
IL-10: Interleukin-10
TNF-alpha: Tumor Necrosis Factor-alpha
TGF-beta: Transforming Growth Factor-beta
CRP: C-reactive protein
BDNF: Brain-Derived Neurotrophic Factor
FGF: Fibroblast Growth Factor
FGF-2: Fibroblast Growth Factor 2
IGF-1: Insulin-Like Growth Factor 1
IGF-1-R: Insulin-Like Growth Factor 1 Receptors
MARK I: MAPK-Activated Protein Kinase 1
MARK II: MAPK-Activated Protein Kinase 2
p-MARK II: Phosphorylation of MAPK-Activated Protein Kinase 2
CaMK-II: Calmodulin Dependent Protein Kinase II
IGFR: Insulin Like Growth Factor Receptors
TrkB: Tropomyosin-Related Kinase B
TrkB-R: Tropomyosin-Related Kinase B Receptors
RIS1: Ras-Induced Senescence 1
PI3Ks: Phosphoinositide 3-Kinases
mTOR: Mechanistic Target Of Rapamycin
p70S6K: 70 kDa Ribosomal S6 Kinase
4EBP: eIF4E-Binding Protein
CREB: cAMP-Response Element Binding Protein
p-CREB: Phosphorylation of cAMP-Response Element Binding Protein
GPx: Glutathione Peroxidases
SOD: Superoxide Dismutase
VEGF: Vascular Endothelial Growth Factor
GFAP: Glial Fibrillary Acidic Protein
GPCRs: G-Protein Coupled Receptors
tPA: Tissue Plasminogen Activator
hGAL: Human Plasma Glycophorin
